# Establishment of MELD-lactate clearance scoring system in predicting death risk of critically ill cirrhotic patients

**DOI:** 10.1186/s12876-022-02351-5

**Published:** 2022-06-03

**Authors:** Xin Li, Man Gong, Shuangnan Fu, Jingjing Zhang, Shanbin Wu

**Affiliations:** 1 Hepatology Department Traditional Chinese Medicine, The Fifth Medical Center of Chinese PLA General Hospital, Beijing, 100039 People’s Republic of China; 2 Department of Gastroenterology, The Second Hospital of Dalian Medical University, No. 467 Zhongshan Road, Shahekou District, Dalian, 116023 Liaoning People’s Republic of China

**Keywords:** Lactate clearance, MELD-ΔLA score, ICU death, Critically ill cirrhotic patients

## Abstract

**Background:**

To develop a scoring system related to the lactate clearance (ΔLA) to predict the mortality risk (MELD-ΔLA) for critically ill cirrhotic patients.

**Methods:**

In this retrospective cohort study, 881 critically ill cirrhotic patients from the Medical Information Mart for Intensive Care (MIMIC-III) database were included eventually. The outcomes of our study were defined as ICU death, 28-day, 90-day and 1-year mortality. Predictors were identified by multivariate Cox analysis to develop the predictive scoring system. The C-index and area under the curve (AUC) of receiver operator characteristic curve (ROC) were used to identify the predicting performance of the MELD-ΔLA, sequential organ failure assessment (SOFA), chronic liver failure-sequential organ failure assessment (CLIF-SOFA), the model for end-stage liver disease (MELD), Child–Pugh, chronic liver failure consortium acute-on-chronic liver failure (CLIF-C ACLF), chronic liver failure consortium-acute decompensation (CLIF-C AD) and MELD-Na scoring systems. Additionally, subgroup analysis was also performed based on whether critically ill cirrhotic patients underwent liver transplantation.

**Results:**

Creatinine, bilirubin, international normalized ratio (INR), lactate first, ΔLA and vasopressors were closely associated with ICU death of liver critically ill cirrhotic patients. The C-index of the MELD-ΔLA in ICU death was 0.768 (95% CI 0.736–0.799) and the AUC for the MELD-ΔLA scoring system in predicting 28-day, 90-day, and 1-year mortality were 0.774 (95% CI 0.743–0.804), 0.765 (95% CI 0.735–0.796), and 0.757 (95% CI 0.726–0.788), suggested that MELD-ΔLA scoring system has a good predictive value than SOFA, CLIF-SOFA, MELD, Child–Pugh, CLIF-C ACLF, CLIF-C AD) and MELD-Na scoring systems. Additionally, the study also confirmed the good predictive value of MELD-ΔLA scoring system for critically ill cirrhotic patients regardless of undergoing liver transplantation.

**Conclusion:**

The developed MELD-ΔLA score is a simple scoring system in predicting the risk of ICU death, 28-day, 90-day and 1-year mortality for critically ill cirrhotic patients, which may have a good predictive performance.

**Supplementary Information:**

The online version contains supplementary material available at 10.1186/s12876-022-02351-5.

## Background

Liver cirrhosis is a major health problem, causing high mortality and economic burden worldwide [1]. A large proportion of liver cirrhosis patients are prone to acute decompensation with organ failure, which need to be admitted to intensive care unit (ICU) [2]. Though liver cirrhosis patients have improved outcomes in ICU over the past decade, the prognosis for these patients still remains poor, with in-hospital mortality rates ranging from 39 to 83% [2]. Therefore, it is essential to carefully evaluate the prognosis of critically ill cirrhotic patients in clinical application, thereby reducing the risk of mortality.

Scoring systems in patients with liver cirrhosis have been widely used in assessing the prognosis of critically ill cirrhotic patients and making clinical decision, such as Child–Pugh score [3], sequential organ failure assessment (SOFA) score [4], chronic liver failure-sequential organ failure assessment (CLIF-SOFA) score [5], the model for end-stage liver disease (MELD) score [6], chronic liver failure consortium acute-on-chronic liver failure (CLIF-C ACLF) score [7], chronic liver failure consortium-acute decompensation (CLIF-C AD) score [7] and MELD-Na score [8]. At present, MELD was considered as an objective and effective system to evaluate the severity of patients than other scores, and was also a common method to quantify the mortality risk of ICU patients with cirrhosis. But some studies also purposed that only creatinine, bilirubin, and international normalized ratio (INR) were contained in the MELD scores, which might affect the assessment of the degree of cirrhosis [9, 10].

Lactate (LA) level is considered as a marker of metabolic changes caused by tissue hypoxia or stress response caused by adrenaline release [11]. A number of clinical studies have concluded that LA level has a good performance in predicting the prognosis of critically cirrhotic patients, suggesting the combination of LA level in the scoring system may improve the predicting performance of death in patients with liver cirrhosis [12, 13]. One study also confirmed that Child–Pugh-LA score, MELD-LA score, CLIF-SOFA-LA score could increase the predicting accuracy for mortality in acute chronic liver failure patients [11]. Recently, the measurements of LA dynamic changes in ICU have been found that significantly superior to static LA measurements in predicting mortality [14, 15]. A decrease in lactate clearance (ΔLA) has been reported to be closely associated with mortality in critically ill patients [16]. ΔLA seems to be more suitable for predicting death in patients with liver cirrhosis.

However, to our knowledge, there were few studies to assess the predictive value of MELD score incorporating ΔLA among critically ill cirrhotic patients. Herein, the aims of our study were: (1) to explore the risk factors associated with the death risk of critically ill cirrhotic patients, and the relationship of ΔLA and ICU death of critically ill cirrhotic patients; (2) to develop a scoring system related to the ΔLA in predicting the mortality risk in critically ill cirrhotic patients (MELD-ΔLA); (3) to compare the predictive performance of the MELD-ΔLA with SOFA, CLIF-SOFA, MELD, Child–Pugh, CLIF-C ACLF, CLIF-C AD and MELD-Na scores; (4) to assess the predictive performance of MELD-ΔLA score for death in critically ill cirrhotic patients with or without undergoing liver transplantation.

## Methods

### Data sources

Data of this retrospective cohort study were obtained from the Medical Information Mart for Intensive Care (MIMIC-III) database (version 1.3). The MIMIC-III is a large, single-center, freely available database, which contained the comprehensive and high-quality medical records of 50,000 patients admitted to ICU at the Beth Israel Deaconess Medical Center between 2001 and 2012 [17, 18].

### Study eligibility criteria

A total of 1840 patients were extracted from the MIMIC-III database 2001–2012 in this retrospective cohort study. Included criteria: ICU patients aged ≥ 18 years old with liver cirrhosis. However, participants were excluded from the study if they met one of the following criteria: (1) due to the data derived from the MIMIC-III database, we found there were some outliers in the age of the population, such as > 300 years of age; (2) patients with the history of liver transplantation; (3) patients without doing two lactate measurements who died within 48 h of ICU admission (Additional file 1: Fig. S1). Due to public availability of MIMIC-III database, with private information of all patients being anonymized, the local ethics committee’s approval was not required.

### Data collection

We collected the baseline characteristics and laboratory parameters of all patients on the first day after ICU admission. The baseline characteristics were included: age (years), gender, insurance type, ICU type, marital status, race, etiology of cirrhosis, acute chronic liver failure (ACLF), characteristics of cirrhosis, therapy. Laboratory parameters included temperature (℃), heart rate (times/min), systolic blood pressure (SBP) and diastolic blood pressure (DBP), mean arterial pressure (MAP), ratio of pulse oxygen saturation to fraction of inspired oxygen (SPO_2_/FiO_2_), ratio of arterial oxygen partial pressure to fraction of inspired oxygen (PaO_2_/FiO_2_), bilirubin (mg/dL), creatinine (mg/dL), international normalized ratio (INR), prothrombin time (PT), albumin, glucose, pH, sodium (mmol/L), potassium (mmol/L), hemoglobin (g/dL), white blood cell (WBC, 10^9^/L) count, chloride, platelet count (PLT, 10^9^/L), lactate first (mmol/L), lactate last (mmol/L), ICU stay time (days). SOFA, CLIF-SOFA, MELD, Child–Pugh, CLIF-C ACLF, CLIF-C AD and MELD-Na.

Liver cirrhosis was defined as advanced liver fibrosis caused by multiple forms of liver disease, including hepatitis and chronic alcoholism [19]. In this study, we used International Classification of Disease (ICD-9) codes to identify critically ill cirrhotic patients (5712, 5715 and 5716) and critically ill cirrhotic patients who underwent liver transplantation (5051 and 5059). Lactate first: lactate of first measurement within 2 days after admission to ICU; lactate last: lactate of last measurement within 2 days after admission to ICU; ΔLA was defined as: (lactate first-lactate last)/lactate first × 100; the MELD score was calculated as following: 9.57 × loge [creatinine (mg/dL)] + 3.78 × loge [bilirubin (mg/dL)] + 11.2 × loge (INR) + 6.43 [18]; the CLIF-C ACLF score was calculated by using the following formula: 10 × (0.33 × CLIF-C organ failure score + 0.04 × Age + 0.63 × ln (WBC)-2 [7]; the CLIF-C ACLF-D score was calculated: CLIF-C ACLF-D score = [(0.03 × Age) + (0.45 × Ascites) + (0.26 × ln (WBC)] − (0.37 × Albumin) + [0.57 × ln (Bilirubin)] + [1.72 × ln (Creatinine)] + 3 × 10 [7]. The MELD-Na score was obtained: MELD-Na = MELD + 1.32 × (137-Na)- [0.033 × MELD × (137-Na)] [20]; the CLIF-SOFA, SOFA and Child–Pugh scores were calculated according to published methods, respectively [21–23].

### Outcomes

The outcomes of our study were defined as ICU death, 28-day, 90-day and 1-year mortality. ICU death was regarded as death before ICU discharge. The 28-day, 90-day and 1-year mortality were defined as deaths within 28 days, 90 days, and 1 year after ICU discharge. The start date of follow-up was the date of the patient’s admission. The follow-up time was 1 year, and once death occurred for patients, the follow-up was terminated. The rate of loss to follow-up in this study was 0%.

### Statistical analysis

The measurement data of the normal distribution were reported as Mean ± Standard Error (Mean ± SE); and the comparison between groups was performed by Student’s t-test. Median and quartile spacing [M (Q1, Q3)] was used to depict the non-normal data; and the comparison between the two groups was analyzed by Mann–Whitney U rank-sum test. The categorical data was described by the number of cases and composition ratio n (%), and were compared by Chi-square or Fisher’s exact test. These missing values were filled by using multiple imputation (Additional file 2: Table S1).

Firstly, all eligible patients were divided into ICU-survival group and ICU-death group based on the occurrence of ICU death, and conducted the difference analysis between two groups. Then we performed the univariate and multivariate COX analyses which aimed to explore the independent predictors related to ICU death for critically ill cirrhotic patients. And we used these related predictors to develop a predictive scoring system (MELD-ΔLA) in predicting the ICU mortality risk among critically ill cirrhotic patients. Next, the C-index and area under the curve (AUC) of receiver operator characteristic curve (ROC) were adopted to compare the predicting performance between developed MELD-ΔLA and other scoring systems (SOFA, CLIF-SOFA, MELD, Child–Pugh, CLIF-C ACLF, CLIF-C AD and MELD-Na scores) for ICU death, 28-day, 90-day and 1-year mortality among critically ill cirrhotic patients. Calibration curves were used to evaluate the calibration of the developed MELD-ΔLA score, and the more closely the two lines fit, indicates a better prediction. Finally, subgroup analysis was performed based on whether critically ill cirrhotic patients underwent liver transplantation, and hazard ratio (HR) and 95% confidence interval (CI) were calculated. The two-sided test was conducted for all statistical analyses. SAS 9.4 software was used for all statistical analyses and R 4.0.2 software was used for drawing. *P* < 0.05 was considered statistically significant. This study followed TRIPOD guidelines for the development of scores for outcome prediction.

## Results

### Baseline characteristics

After excluded patients who were over 300 years of age (n = 15), had the history of liver transplant (n = 140), and were not made two lactate measurements or died within 48 h of ICU admission (n = 804), a total of 881 eligible patients were enrolled eventually in this study, with an average age of 58.65 ± 11.57 years old. They were divided into ICU-survival group (n = 628) and ICU-death group (n = 253), respectively. As shown in Table 1, there were some variables (such as ICU type, lactate first, lactate last, ΔLA, et al.) with significant differences between two groups (*P* < 0.05).

**Table 1:** Baseline characteristics and laboratory parameters of all patients

Variables	Total (n = 881)	ICU-survival group (n = 628)	ICU-death group (n = 253)	Statistics	*P*
Age, years, Mean ± SD	58.65 ± 11.57	58.33 ± 11.20	59.47 ± 12.43	t = − 1.260	0.207
*Gender, n (%)*				χ^2^ = 1.734	0.188
Female	288 (32.69)	197 (31.37)	91 (35.97)		
Male	593 (67.31)	431 (68.63)	162 (64.03)		
*Insurance, n (%)*				χ^2^ = 0.725	0.696
Medicare	378 (42.91)	271 (43.15)	107 (42.29)		
Others	203 (23.04)	140 (22.29)	63 (24.90)		
Private	300 (34.05)	217 (34.55)	83 (32.81)		
*ICU type, n (%)*				χ^2^ = 11.233	0.004
MICU	486 (55.16)	327 (52.07)	159 (62.85)		
CCU/CSRU	95 (10.78)	66 (10.51)	29 (11.46)		
SICU/TSICU	300 (34.05)	235 (37.42)	65 (25.69)		
*Marital, n (%)*				χ^2^ = 0.955	0.812
Married	389 (44.15)	272 (43.31)	117 (46.25)		
Single	316 (35.87)	226 (35.99)	90 (35.57)		
Widowed	52 (5.90)	38 (6.05)	14 (5.53)		
Divorced/separated	124 (14.07)	92 (14.65)	32 (12.65)		
*Race, n (%)*				χ^2^ = 5.776	0.217
White	688 (78.09)	490 (78.03)	198 (78.26)		
Black	76 (8.63)	49 (7.80)	27 (10.67)		
Asian	26 (2.95)	17 (2.71)	9 (3.56)		
Hispanic	48 (5.45)	40 (6.37)	8 (3.16)		
Others	43 (4.88)	32 (5.10)	11 (4.35)		
*ACLF, n (%)*				χ^2^ = 62.746	< 0.001
No	325 (36.89)	283 (45.06)	42 (16.60)		
Yes	556 (63.11)	345 (54.94)	211 (83.40)		
*Pathogenesis, n (%)*				χ^2^ = 2.252	0.324
Alcoholic	426 (48.35)	298 (47.45)	128 (50.59)		
No alcoholic	438 (49.72)	320 (50.96)	118 (46.64)		
Biliary	17 (1.93)	10 (1.59)	7 (2.77)		
Hepatic encephalopathy, n (%)	224 (25.43)	159 (25.32)	65 (25.69)	χ^2^ = 0.013	0.908
Ascites, n (%)	281 (31.90)	200 (31.85)	81 (32.02)	χ^2^ = 0.002	0.961
Hepatocerebral syndrome, n (%)	153 (17.37)	100 (15.92)	53 (20.95)	χ^2^ = 3.173	0.075
RRT, n (%)	81 (9.19)	46 (7.32)	35 (13.83)	χ^2^ = 9.152	0.002
Vasopressors, n (%)	290 (32.92)	124 (19.75)	166 (65.61)	χ^2^ = 171.822	< 0.001
Ventilation, n (%)	369 (41.88)	261 (41.56)	108 (42.69)	χ^2^ = 0.094	0.759
Transplantation, n (%)	106 (12.03)	104 (16.56)	2 (0.79)	χ^2^ = 42.375	< 0.001
Temperature, ℃, Mean ± SD	36.66 ± 0.86	36.70 ± 0.84	36.54 ± 0.89	t = 2.590	0.010
Heart rate, times/min, Mean ± SD	94.90 ± 18.76	93.51 ± 18.51	98.34 ± 18.97	t = − 3.480	< 0.001
SBP, Mean ± SD	119.88 ± 20.35	121.07 ± 20.52	116.91 ± 19.66	t = 2.760	0.006
DBP, M (Q1, Q3)	63.00 (53.00, 72.00)	63.00 (54.50, 72.00)	61.00 (50.00, 71.00)	Z = − 2.346	0.019
MAP, Mean ± SD	77.37 ± 17.96	78.39 ± 17.32	74.83 ± 19.28	t = 2.550	0.011
SPO_2_/FiO_2_, M (Q1, Q3)	100.00 (98.00, 194.00)	100.00 (99.00, 198.00)	100.00 (96.00, 178.00)	Z = − 3.917	< 0.001
PaO_2_/FiO_2_, M (Q1, Q3)	278.00 (170.00, 395.00)	310.00 (183.33, 418.00)	220.00 (136.67, 322.00)	Z = − 6.493	< 0.001
Bilirubin, mg/dL, M (Q1, Q3)	2.50 (1.20, 6.20)	2.10 (1.00, 5.00)	4.00 (1.80, 10.30)	Z = 6.223	< 0.001
Creatinine, mg/dL, M (Q1, Q3)	1.20 (0.90, 2.20)	1.10 (0.80, 1.90)	1.60 (1.00, 2.80)	Z = 5.606	< 0.001
INR, M (Q1, Q3)	1.70 (1.40, 2.20)	1.60 (1.30, 2.00)	2.10 (1.70, 2.60)	Z = 10.699	< 0.001
PT, M (Q1, Q3)	17.60 (14.90, 21.40)	16.80 (14.60, 20.10)	19.70 (16.30, 24.20)	Z = 6.917	< 0.001
Albumin, Mean ± SD	2.86 ± 0.66	2.93 ± 0.66	2.67 ± 0.61	t = 5.540	< 0.001
Glucose, M (Q1, Q3)	118.00 (97.00, 155.00)	120.00 (99.00, 158.50)	116.00 (92.00, 146.00)	Z = − 1.858	0.063
PH, Mean ± SD	7.36 ± 0.11	7.37 ± 0.10	7.33 ± 0.14	t = 4.790	< 0.001
Sodium, mmol/L, Mean ± SD	135.62 ± 6.78	136.04 ± 6.31	134.58 ± 7.75	t = 2.670	0.008
Potassium, mmol/L, Mean ± SD	4.39 ± 0.97	4.36 ± 0.94	4.47 ± 1.03	t = − 1.510	0.131
Hemoglobin, g/dL, Mean ± SD	10.84 ± 2.39	10.93 ± 2.41	10.64 ± 2.32	t = 1.650	0.099
WBC, 10^9^/L, M (Q1, Q3)	8.90 (5.70, 13.60)	8.40 (5.40, 12.55)	10.30 (6.30, 14.90)	Z = 3.168	0.002
Chloride, Mean ± SD	102.17 ± 7.74	102.68 ± 7.31	100.92 ± 8.60	t = 2.850	0.005
PLT, 10^9^/L, M (Q1, Q3)	114.00 (72.00, 176.00)	121.00 (76.00, 179.00)	105.00 (66.00, 170.00)	Z = − 2.628	0.009
Lactate last, mmol/L, M (Q1, Q3)	1.90 (1.40, 3.00)	1.70 (1.30, 2.30)	3.20 (1.90, 6.40)	Z = 12.117	< 0.001
Lactate first, mmol/L, M (Q1, Q3)	2.40 (1.60, 4.00)	2.10 (1.40, 3.40)	3.30 (2.20, 5.90)	Z = 8.680	< 0.001
ΔLA, %, M (Q1, Q3)	16.00 (− 22.00, 45.00)	18.00 (− 14.00, 48.00)	2.00 (− 53.00, 39.00)	Z = − 4.095	< 0.001
ICU stay time, days, M (Q1, Q3)	3.82 (2.01, 8.58)	3.60 (2.01, 7.44)	4.72 (2.00, 11.49)	Z = 2.402	0.016
SOFA	9.00 (7.00,12.00)	8.00 (6.00,10.00)	12.00 (9.00,15.00)	Z = 11.758	< 0.001
CLIF-SOFA	8.00 (6.00,11.00)	7.00 (5.00,10.00)	11.00 (8.00,13.00)	Z = 11.402	< 0.001
MELD	18.72 (11.32,27.23)	16.34 (10.10,24.74)	24.89 (17.01,31.89)	Z = 8.445	< 0.001
Child–Pugh	9.17 ± 2.44	8.84 ± 2.49	9.98 ± 2.10	t = − 6.94	< 0.001
CLIF-C ACLF	48.80 ± 8.80	47.29 ± 7.98	51.28 ± 9.52	T = − 5.10	< 0.001
CLIF-C AD	36.66 ± 1.25	31.54 ± 1.18	32.43 ± 1.48	T = − 3.71	< 0.001
MELD-Na	25.08 (16.04,33.81)	22.03 (14.31,31.29)	32.64 (24.04,38.65)	Z = 9.804	< 0.001

ICU, intensive care unit; MICU, medical intensive care unit; CCU, coronary care unit; CSRU, cardiac surgery care unit; ICU, surgical intensive care unit; TSICU, traumatic surgical intensive care unit; ACLF, acute chronic liver failure; RRT, renal replacement therapy; SBP, systolic blood pressure; DBP, diastolic blood pressure; MAP, mean arterial pressure; PaO_2_, arterial oxygen partial pressure; FiO_2_, fraction of inspired oxygen; SPO_2_, pulse oxygen saturation; INR, international normalized ratio; PT, prothrombin time; PLT, platelet count; WBC, white blood cell count; ΔLA, lactate clearance; SOFA, sequential organ failure assessment; CLIF-SOFA, chronic liver failure-sequential organ failure assessment; MELD, model for end-stage liver disease; CLIF-C ACLF, chronic liver failure consortium acute-on-chronic liver failure score; CLIF-C AD, chronic liver failure consortium-acute decompensation

### Predictor selection of ICU death in critically ill cirrhotic patients

Table 2 suggests that the effects of ACLF, RRT, vasopressors, transplant, temperature, SBP, DBP, MAP, PaO_2_/FiO_2_, PaO_2_/FiO_2_, bilirubin, creatinine, INR, PT, albumin, pH, sodium, potassium, WBC, chloride, lactate first, lactate last and ΔLA on ICU death among patients with critically ill cirrhotic had significant differences (*P* < 0.05). The variables with significant differences in univariate COX analysis were incorporated into multivariate COX analysis and screened step by step. As illustrated in Table 3, creatinine, bilirubin, INR, lactate first, ΔLA and vasopressors were closely associated with ICU death for critically ill cirrhotic patients. For each 1 mg/dL increase in creatinine, bilirubin, INR and lactate first, the risk of ICU death increased 0.075-fold (HR = 1.075, 95% CI 1.004–1.151), 0.022-fold (HR = 1.022, 95% CI 1.008–1.035), 0.102-fold (HR = 1.102, 95% CI 1.011–1.201) and 0.142-fold (HR = 1.142, 95% CI 1.098–1.187), separately among liver cirrhosis patients. Similarly, the risk of ICU death in critically ill cirrhotic patients with vasopressors was 2.560 times higher than those without the using history of vasopressors (HR = 2.560, 95% CI 1.959–3.345). Additionally, it should be noticed that ΔLA (per 1% increase) reduced the risk of ICU death in critically ill cirrhotic patients by 0.341-fold (HR = 0.659, 95% CI 0.610–0.712). Additional file 3: Fig. S3 shows the survival curves dependent on the ΔLA. We took the median of Lactate changes as a cut-off. Compared with the ΔLA < 16.22%, the survival rate of ΔLA ≥ 16.12% was better.

**Table 2:** Univariate COX analysis for the potential predictors of ICU death in liver cirrhosis patients

Variables	β	SE	χ^2^	*P*	HR	95% CI	
						Lower	Upper
Age	0.007	0.005	1.668	0.196	1.007	0.997	1.017
Gender (Male)	− 0.133	0.131	1.021	0.312	0.876	0.677	1.133
Insurance							
Medicare					Ref		
Others	0.062	0.159	0.151	0.697	1.064	0.779	1.453
Private	0.045	0.147	0.093	0.761	1.046	0.784	1.394
ICU type							
MICU					Ref		
CCU/CSRU	− 0.219	0.202	1.175	0.278	0.803	0.540	1.194
SICU/TSICU	− 0.368	0.147	6.215	0.013	0.692	0.519	0.924
Marital status							
Married					Ref		
Single	0.087	0.141	0.383	0.536	1.091	0.827	1.440
Widowed	0.049	0.284	0.030	0.863	1.050	0.602	1.831
Divorced/separated	0.061	0.200	0.092	0.761	1.063	0.717	1.574
Race							
White					Ref		
Black	0.236	0.206	1.319	0.251	1.267	0.846	1.896
Asian	0.759	0.343	4.890	0.027	2.136	1.090	4.187
Hispanic	− 0.443	0.361	1.506	0.220	0.642	0.316	1.303
Others	− 0.286	0.310	0.851	0.356	0.751	0.409	1.380
ACLF (Yes)	0.704	0.171	17.032	< 0.001	2.021	1.447	2.823
Pathogenesis							
Alcoholic					Ref		
No alcoholic	0.233	0.389	0.360	0.549	1.263	0.589	2.705
Biliary	0.127	0.129	0.979	0.322	1.136	0.883	1.462
Hepatic encephalopathy (Yes)	− 0.135	0.144	0.880	0.348	0.874	0.659	1.159
Ascites (Yes)	− 0.026	0.135	0.036	0.849	0.975	0.748	1.270
Hepatocerebral syndrome (Yes)	0.159	0.155	1.050	0.306	1.172	0.865	1.587
RRT (Yes)	0.389	0.183	4.541	0.033	1.476	1.032	2.111
Vasopressors (Yes)	1.175	0.133	77.955	< 0.001	3.239	2.495	4.205
Ventilation (Yes)	− 0.128	0.127	1.012	0.314	0.880	0.685	1.129
Transplantation (Yes)	− 2.511	0.710	12.494	< 0.001	0.081	0.020	0.327
Temperature	− 0.216	0.076	8.147	0.004	0.806	0.695	0.935
Heart rate	0.003	0.003	1.061	0.303	1.003	0.997	1.009
SBP	− 0.008	0.003	5.315	0.021	0.992	0.986	0.999
DBP	< 0.001	0.000	5.498	0.019	1.000	1.000	1.001
MAP	− 0.008	0.004	4.727	0.030	0.992	0.985	0.999
SPO_2_/FiO_2_	− 0.002	0.001	4.847	0.028	0.998	0.996	1.000
PaO_2_/FiO_2_	− 0.002	0.000	19.165	< 0.001	0.998	0.997	0.999
Bilirubin	0.027	0.006	23.490	< 0.001	1.028	1.016	1.039
Creatinine	0.124	0.031	15.708	< 0.001	1.132	1.065	1.203
INR	0.156	0.028	30.121	< 0.001	1.169	1.105	1.235
PT	0.028	0.005	29.509	< 0.001	1.028	1.018	1.039
Albumin	− 0.362	0.094	14.827	< 0.001	0.696	0.579	0.837
Glucose	< 0.001	0.001	0.253	0.615	1.001	0.998	1.003
PH	− 2.608	0.526	24.571	< 0.001	0.074	0.026	0.207
Sodium	− 0.043	0.009	22.916	< 0.001	0.958	0.941	0.975
Potassium	0.173	0.062	7.936	0.005	1.189	1.054	1.342
Hemoglobin	− 0.018	0.027	0.469	0.493	0.982	0.931	1.035
WBC	0.022	0.008	7.180	0.007	1.023	1.006	1.040
Chloride	− 0.032	0.008	15.679	< 0.001	0.969	0.954	0.984
PLT	− 0.001	0.001	2.599	0.107	0.999	0.997	1.000
Lactate last	0.263	0.015	328.456	< 0.001	1.301	1.265	1.339
Lactate first	0.117	0.018	43.252	< 0.001	1.124	1.086	1.164
ΔLA	− 0.350	0.038	85.886	< 0.001	0.704	0.654	0.759

ICU, intensive care unit; MICU, medical intensive care unit; CCU, coronary care unit; CSRU, cardiac surgery care unit; ICU, surgical intensive care unit; TSICU, traumatic surgical intensive care unit; ACLF, acute chronic liver failure; RRT, renal replacement therapy; SBP, systolic blood pressure; DBP, diastolic blood pressure; MAP, mean arterial pressure; PaO_2_, arterial oxygen partial pressure; FiO_2_, fraction of inspired oxygen; SPO_2_, pulse oxygen saturation; INR, international normalized ratio; PT, prothrombin time; PLT, platelet count; WBC, white blood cell count; ΔLA, lactate clearance;

**Table 3:** Multivariate COX analysis for the predictors of ICU death in liver cirrhosis patients

Variables	β	SE	χ^2^	*P*	HR	95% CI	
						Lower	Upper
Creatinine	0.072	0.035	4.275	0.039	1.075	1.004	1.151
Bilirubin	0.021	0.007	10.107	0.001	1.022	1.008	1.035
INR	0.097	0.044	4.877	0.027	1.102	1.011	1.201
Lactate first	0.132	0.020	44.389	< 0.001	1.142	1.098	1.187
ΔLA	− 0.417	0.039	112.266	< 0.001	0.659	0.610	0.712
Vasopressors (Yes)	0.940	0.136	47.440	< 0.001	2.560	1.959	3.345

INR, international normalized ratio; ΔLA, lactate clearance; HR, hazard ratio; CI, confidence interval

### Establishment and performance of the MELD-ΔLA scoring system

**Fig. 1:**
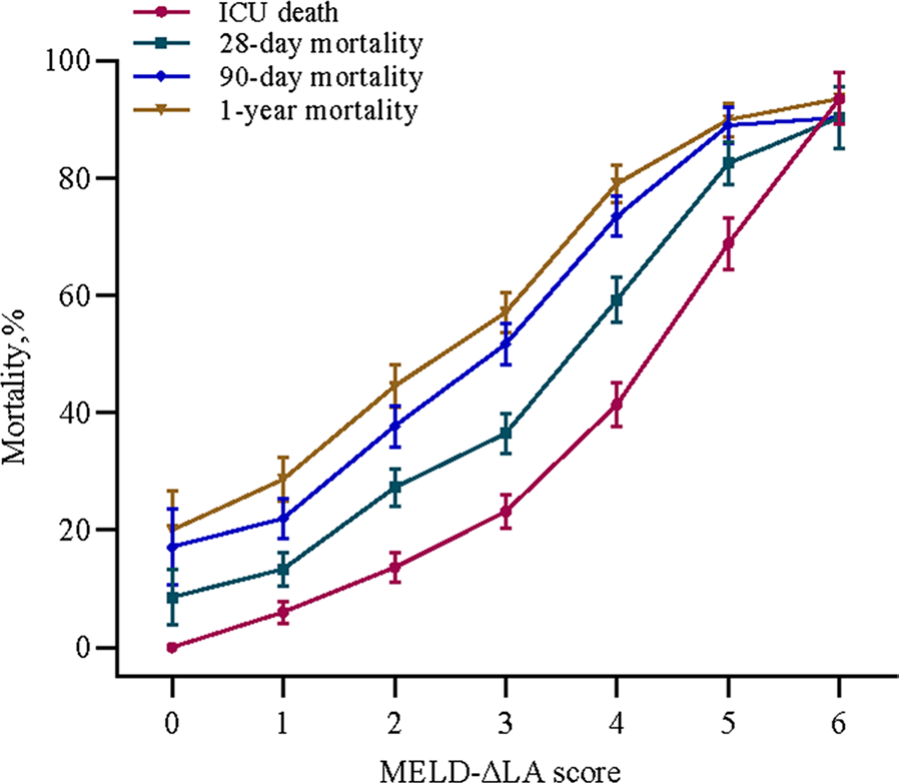
The association of MELD-ΔLA score and ICU death, 28-day, 90-day and 1-year mortality in liver cirrhosis patients

MELD-ΔLA score was generated by the optimal cutoff point for each factor determined via the Youden index. MELD-ΔLA score was composed of creatinine, bilirubin, INR, lactate first, ΔLA and the using history of vasopressors, and detailed information was given in Table 4. Furthermore, we also calculated a combined formula based on the formula of a regression model with metric variables as following: (Combined formula = h_0_(t) × e (Creatinine × 0.072+ Bilirubin × 0.021 + INR × 0.097+ Lactate first × 0.132 − ΔLA × 0.417 + Vasopressors (Yes) × 0.940). And we compared the predicting performance of combined formula and MELD-ΔLA score (Table 5). Although the C-indexes of the combined formula in ICU death was 0.789 (95% CI 0.756–0.823), and the AUC for the combined formula in predicting 28-day, 90-day, and 1-year mortality were 0.800 (95% CI 0.771–0.830), 0.785 (95% CI 0.755–0.814), and 0.762 (95% CI 0.731–0.793), respectively, which were higher than MELD-ΔLA score, there was no significant difference for combined formula, thus we cannot obtain a conclusion that the predicting performance of combined formula was higher than MELD-ΔLA score. Additionally, MELD-ΔLA score was a more simple and convenient scoring system for clinical application than the combined formula. Figure 1 also indicated that the relation of MELD-ΔLA score and ICU death, 28-day, 90-day and 1-year mortality among critically ill cirrhotic patients, with a positive correlation. Additionally, Additional file 4: Fig. S4 presents a survival figure of using the MELD-ΔLA score, and the result indicated that the higher the MELD-ΔLA score, the worse the survival probability.

**Table 4:** Establishment of the MELD-ΔLA scoring system

Variables	Score	
	0	1
Creatinine, mg/dL	< 1.2	≥ 1.2
Bilirubin, mg/dL	< 2.0	≥ 2.0
INR	< 1.7	≥ 1.7
Lactate first, mmol/L	< 2.6	≥ 2.6
ΔLA, %	≥ 4.0	< 4.0
Vasopressors	No	Yes

INR, international normalized ratio; ΔLA, lactate clearance

**Table 5:** Validation for the performance of MELD-ΔLA scoring system in critically ill cirrhotic patients

Score systems	ICU death		28-day mortality		90-day mortality		1-year mortality	
	C index (95% CI)	*P*	AUC (95% CI)	*P*	AUC (95% CI)	*P*	AUC (95% CI)	*P*
*Total*								
MELD-ΔLA	0.768 (0.736–0.799)	–	0.774 (0.743–0.804)	–	0.765 (0.735–0.796)	–	0.757 (0.726–0.788)	–
ACLF patients	0.725 (0.686–0.764)	–	0.718 (0.677–0.759)	–	0.711 (0.668–0.754)	–	0.702 (0.657–0.746)	–
Non-ACLF patients	0.861 (0.812–0.910)	–	0.775 (0.712–0.838)	–	0.736 (0.677–0.795)	–	0.722 (0.665–0.779)	–
SOFA	0.715 (0.675–0.755)	0.040	0.680 (0.644–0.717)	< 0.001	0.654 (0.618–0.689)	< 0.001	0.634 (0.598–0.670)	< 0.001
CLIF-SOFA	0.722 (0.685–0.758)	0.047	0.759 (0.727–0.790)	0.238	0.756 (0.724–0.787)	0.430	0.734 (0.702–0.767)	0.072
MELD	0.671 (0.634–0.708)	< 0.001	0.716 (0.682–0.750)	< 0.001	0.718 (0.684–0.751)	< 0.001	0.709 (0.674–0.743)	< 0.001
Child–Pugh	0.617 (0.580–0.654)	< 0.001	0.681 (0.646–0.716)	< 0.001	0.696 (0.661–0.730)	< 0.001	0.687 (0.652–0.723)	< 0.001
Combined formula	0.789 (0.756–0.823)	0.350	0.800 (0.771–0.830)	0.233	0.785 (0.755–0.814)	0.366	0.762 (0.731–0.793)	0.824
CLIF-C ACLF (for ACLF patients)	0.652 (0.608–0.696)	< 0.001	0.696 (0.652–0.739)	0.425	0.691 (0.647–0.736)	0.468	0.684 (0.637–0.730)	0.532
CLIF-C AD (for non-ACLF patients)	0.717 (0.619–0.816)	0.011	0.705 (0.635–0.775)	0.074	0.688 (0.625–0.751)	0.181	0.700 (0.642–0.759)	0.513
MELD-Na	0.708 (0.671–0.746)	0.017	0.744 (0.710–0.777)	0.204	0.731 (0.698–0.764)	0.143	0.712 (0.678–0.746)	0.057
*Liver transplant*								
MELD-ΔLA	0.887 (0.749–0.999)	–	0.788 (0.698–0.861)	–	0.778 (0.687–0.853)	–	0.698 (0.601–0.783)	–
ACLF patients	–	–	0.896 (0.733–1.000)	–	0.757 (0.466–1.000)	–	0.779 (0.567–0.992)	–
Non-ACLF patients	–	–	0.682 (0.057–1.000)	–	0.682 (0.057–1.000)	–	0.604 (0.300–0.907)	–
SOFA	0.514 (0.361–0.667)	< 0.001	0.571 (0.471–0.667)	0.054	0.572 (0.472–0.668)	0.025	0.518 (0.419–0.617)	0.381
CLIF-SOFA	0.577 (0.432–0.722)	0.002	0.555 (0.455–0.652)	< 0.001	0.611 (0.511–0.704)	0.037	0.524 (0.425–0.622)	0.001
MELD	0.606 (0.353–0.859)	0.044	0.517 (0.418–0.615)	0.013	0.574 (0.474–0.670)	0.053	0.504 (0.405–0.602)	0.004
Child–Pugh	0.648 (0.463–0.833)	0.042	0.565 (0.465–0.661)	0.148	0.633 (0.534–0.724)	0.298	0.559 (0.460–0.656)	0.123
Combined formula	–	–	0.971 (0.911–1.000)	< 0.001	0.872 (0.704–1.000)	0.281	0.818 (0.610–1.000)	0.264
CLIF-C ACLF (for ACLF patients)	–	–	0.861 (0.713–1.000)	0.650	0.610 (0.113–1.000)	0.563	0.574 (0.212–0.935)	0.269
CLIF-C AD (for non-ACLF patients)	–	–	0.674 (0.293–1.000)	0.967	0.674 (0.293–1.000)	–	0.500 (0.239–0.761)	0.438
MELD-Na	–	–	0.525 (0.181–0.868)	0.136	0.560 (0.247–0.874)	0.174	0.551 (0.324–0.778)	0.209
*Non-liver transplant*								
MELD-ΔLA	0.758 (0.726–0.790)	–	0.767 (0.735–0.796)	–	0.763 (0.731–0.792)	–	0.757 (0.725–0.787)	–
ACLF patients	0.723 (0.684–0.762)	–	0.721 (0.679–0.764)	–	0.722 (0.676–0.768)	–	0.713 (0.665–0.762)	–
Non-ACLF patients	0.847 (0.794–0.900)	–	0.769 (0.703–0.836)	–	0.728 (0.664–0.791)	–	0.720 (0.659–0.782)	–
SOFA	0.716 (0.677–0.755)	0.048	0.699 (0.665–0.731)	< 0.001	0.680 (0.646–0.713)	< 0.001	0.666 (0.631–0.699)	< 0.001
CLIF-SOFA	0.716 (0.679–0.753)	0.042	0.755 (0.723–0.785)	0.392	0.755 (0.723–0.785)	0.574	0.735 (0.702–0.766)	0.105
MELD	0.673 (0.636–0.710)	0.001	0.729 (0.696–0.760)	0.008	0.738 (0.706–0.769)	0.086	0.736 (0.704–0.767)	0.149
Child–Pugh	0.616 (0.579–0.653)	< 0.001	0.682 (0.648–0.715)	< 0.001	0.703 (0.670–0.735)	< 0.001	0.698 (0.664–0.730)	< 0.001
Combined formula	0.781 (0.748–0.814)	–	0.809 (0.778–0.840)	0.062	0.801 (0.771–0.832)	0.086	0.778 (0.745–0.811)	0.366
CLIF-C ACLF (for ACLF patients)	0.646 (0.603–0.689)	0.005	0.693 (0.648–0.738)	0.317	0.688 (0.641–0.736)	0.238	0.681 (0.630–0.731)	0.295
CLIF-C AD (for non-ACLF patients)	0.698 (0.596–0.800)	0.006	0.686 (0.611–0.762)	0.045	0.666 (0.597–0.736)	0.109	0.692 (0.627–0.757)	0.447
MELD-Na	0.716 (0.679–0.753)	0.090	0.771 (0.738–0.804)	0.863	0.770 (0.737–0.802)	0.763	0.760 (0.726–0.794)	0.899

CI, confidence interval; AUC, area under the curve; ICU, intensive care unit; SOFA, sequential organ failure assessment; CLIF-SOFA, chronic liver failure-sequential organ failure assessment; MELD, model for end-stage liver disease; AUC, area under the curve; MELD-ΔLA, model for end-stage liver disease-lactate clearance; ACLF, acute chronic liver failure; CLIF-C ACLF, chronic liver failure consortium acute-on-chronic liver failure score; CLIF-C AD, chronic liver failure consortium-acute decompensation; Combined formula represent the a formula of a regression model with metric variables

**Fig. 2:**
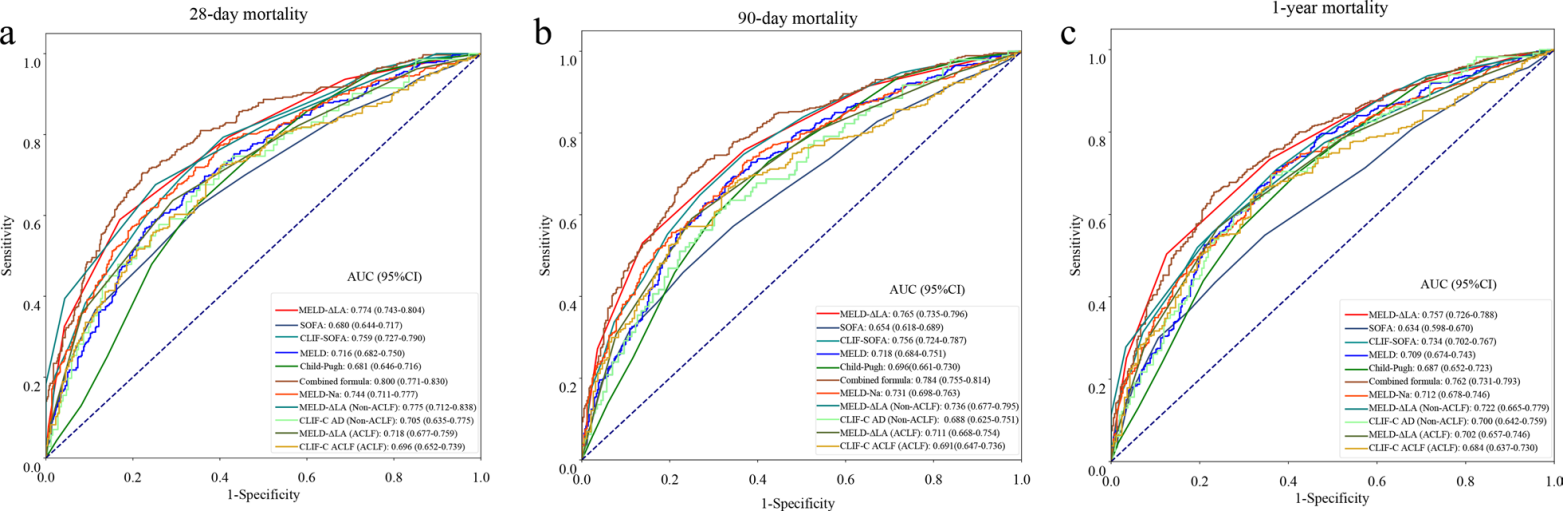
ROC curves of (a) 28-day, (b) 90-day, and (c) 1-year death for MELD-ΔLA score

Importantly, we compared the predicting performance of MELD-ΔLA and SOFA, CLIF-SOFA, MELD, Child–Pugh, CLIF-C ACLF, CLIF-C AD and MELD-Na scoring systems for ICU death, 28-day, 90-day and 1-year mortality among all critically ill cirrhotic patients (Table 5). The C-indexes of the MELD-ΔLA, SOFA, CLIF-SOFA, MELD, Child–Pugh, and MELD-Na in ICU death were 0.768 (95% CI 0.736–0.799), 0.715 (95% CI 0.675–0.755), 0.722 (95% CI 0.685–0.758), 0.671 (95% CI 0.634–0.708), 0.617 (95% CI 0.580–0.654) and 0.708 (95% CI 0.671–0.746), separately; the AUC for the MELD-ΔLA scores in predicting 28-day, 90-day, and 1-year mortality were 0.774 (95% CI 0.743–0.804), 0.765 (95% CI 0.735–0.796), and 0.757 (95% CI 0.726–0.788), respectively, which were obviously higher than SOFA, CLIF-SOFA, MELD, Child–Pugh and MELD-Na scoring systems (Fig. 2). Also, for patients with ACLF, the C-indexes of the MELD-ΔLA score and CLIF-C ACLF score in ICU death were 0.725 (95% CI 0.686–0.764) and 0.652 (95% CI 0.608–0.696), respectively; and the AUC for the CLIF-C ACLF score in predicting 28-day, 90-day, and 1-year mortality were 0.696 (95% CI 0.652–0.739), 0.691 (95% CI 0.647–0.736), and 0.684 (95% CI 0.637–0.730), separately, which were lower than MELD-ΔLA score. Similarly, for patients without ACLF, the C-indexes of the MELD-ΔLA score and CLIF-C AD score in ICU death were 0.861 (95% CI 0.812–0.910) and 0.717 (95% CI 0.619–0.816), respectively; and the AUC for the CLIF-C AD score in predicting 28-day, 90-day, and 1-year mortality were 0.705 (95% CI 0.635–0.775), 0.688 (95% CI 0.625–0.751), and 0.700 (95% CI 0.642–0.759), respectively, which were also lower than MELD-ΔLA score. In short, these results indicated that the predicting performance of developed MELD-ΔLA score was higher SOFA score, CLIF-SOFA score, MELD score, Child–Pugh score, MELD-Na score, the CLIF-C ACLF score in patients with ACLF, and the CLIF-C AD score in patients without ACLF.

Furthermore, the calibration curves also revealed a relatively high degree of agreement between the prediction results of MELD-ΔLA scoring system and the actual results (Additional file 5: Fig. S5). These results implied that MELD-ΔLA score has a good discrimination in predicting the risk of ICU death, 28-day, 90-day and 1-year mortality for critically ill cirrhotic patients.

### Validation for the performance of scoring system in different subgroups based on whether patients with critically ill cirrhotic underwent liver transplant

In this study, we also carried out a subgroup analysis based on whether critically ill cirrhotic patients underwent liver transplantation (Table 5). As shown in Table 5, the C-indexes of the MELD-ΔLA scoring system were 0.887 (95% CI 0.749–0.999) in critically ill cirrhotic patients underwent liver transplantation and 0.758 (95% CI 0.726–0.790) in critically ill cirrhotic patients without liver transplantation. In addition, the AUC for the MELD-ΔLA scoring system at predicting 28-day, 90-day, and 1-year mortality were higher than other scoring systems (SOFA, CLIF-SOFA, MELD, Child–Pugh, CLIF-C ACLF, CLIF-C AD and MELD-Na), regardless of whether critically ill cirrhotic patients had undergone liver transplantation. These results confirmed a good predictive value of MELD-ΔLA score regardless of undergoing liver transplantation for critically ill cirrhotic patients.

## Discussion

In this retrospective cohort study, we reported several key factors regarding the risk of ICU death among critically ill cirrhotic patients and expounded the negative correlation between ΔLA and the risk of ICU death among critically ill cirrhotic patients. More importantly, our study developed an objective and simpler prediction score (MELD-ΔLA) to assess the risk of ICU death, 28-day, 90-day and 1-year mortality among critically ill cirrhotic patients, and there was a significantly positive association between MELD-ΔLA score and ICU death, 28-day, 90-day and 1-year mortality in critically ill cirrhotic patients. Additionally, the findings illustrated that MELD-ΔLA score has a better predictive value than other scores (SOFA, CLIF-SOFA, MELD, Child–Pugh, CLIF-C ACLF, CLIF-C AD and MELD-Na) among critically ill cirrhotic patients. Not only that, the MELD-ΔLA score also showed the good predictive value for critically ill cirrhotic patients who had or had not undergone liver transplantation.

MELD-ΔLA score mainly contained six predictors of creatinine, bilirubin, INR, lactate first, ΔLA and the using history of vasopressors. The levels of creatinine, bilirubin, INR were closely associated with the risk of ICU death among critically ill cirrhotic patients, which were consistent with previous studies [6, 24, 25]. It’s worth paying attention to ΔLA plays a vital role in the MELD-ΔLA scoring system among critically ill cirrhotic patients. Recently, lactate level has been proved to assess the severity of disease, which has become an effective biomarker in clinical diagnosis [10]. There was a high lactate level for most patients with chronic liver disease, which the reason may be tissue hypoperfusion of critically ill patients and decreased ΔLA in advanced liver disease [26]. One study has showed that lactate level not only reflected the severity of organ failure, but was also an independently risk factor for short-term mortality of critically ill cirrhotic patients [12]. Several studies have incorporated lactate level into scoring systems to better predict death in patients with liver cirrhosis [27, 28]. Sarmast et al. developed a MELD-Lactate model and reported that MELD-Lactate model could be used to identify patients with chronic liver disease for the risk of ICU mortality [9]. Nevertheless, simply measuring lactate level at one time may be not accurately reflect the dynamic oxygenation status of tissues and the severity of disease, thereby, more attention should be paid to the role of ΔLA [29, 30]. To date, most researches only focused on the development of lactate level in liver cirrhosis patients [12, 27], there was few studies to investigate the influence of ΔLA in the scoring system for the ICU mortality. To our knowledge, this is the first study conducted to develop a MELD-ΔLA score to predict the risk of ICU death and 28-day, 90-day and 1-year death among critically ill cirrhotic patients; the decreased of ΔLA was distinctly related to higher risk of ICU mortality among critically ill cirrhotic patients.

The predictive factors of the developing MELD-ΔLA scoring system could be easily obtained in critically ill cirrhotic patients, which suggested the scoring system would be simpler and more convenient for the clinical practice. Besides, the study also found that MELD-ΔLA scoring system expressed an advanced diagnostic discrimination than other scoring systems (SOFA, CLIF-SOFA, MELD, Child–Pugh, CLIF-C ACLF, CLIF-C AD and MELD-Na) in ICU death and 28-day, 90-day and 1-year mortality for critically ill cirrhotic patients. Likewise, the MELD-ΔLA scoring system has also been demonstrated a good predictive ability for critically ill cirrhotic patients who were with or without undergoing liver transplantation. In general, the developed MELD-ΔLA score is a simple, intuitive, and objective scoring system to help clinician in better predicting the risk of ICU death and 28-day, 90-day and 1-year mortality for critically ill cirrhotic patients than other scoring systems.

The major strength of our study is the development of MELD-ΔLA scoring system which makes it easier for clinicians to assess the risk of death among critically ill cirrhotic patients. However, this study also has some limitations. Firstly, the sample size was not large enough with a retrospective cohort study, which might have bias for the result of our study. Secondly, because of all data of this study derived from the MIMIC-III database, we could not get the specific time of lactate measurement for all patients. Thirdly, the developed MELD-ΔLA score has a better predictive value than other scoring systems, which was only applicable to critically ill cirrhotic patients. We cannot be sure if MELD-ΔLA score has similar predictive power among other populations. Finally, there was a lack of external validation to assess the predictive ability of this scoring system. These should be cautious in interpreting the results. Hence, further studies should be required to validate the results of the present study, promoting the introduction of this scoring system into clinical practice.

## Conclusion

In summary, the developed MELD-ΔLA score is a simple scoring system to assess the risk of ICU death, 28-day, 90-day and 1-year death among critically ill cirrhotic patients, which might have a better predictive value compared to SOFA, CLIF-SOFA, MELD, Child–Pugh, CLIF-C ACLF, CLIF-C AD and MELD-Na scores.

## Supplementary Information

**Additional** **file 1.** **Supplemental Figure 1.** The consort figure of extracted patient.

**Additional** **file 2.** **Supplemental Table 1.** The sensitivity analysis of missing data before and after interpolation.

**Additional** **file** **3.** **Supplemental Figure 2.** The survival curves dependent on the lactate changes.

**Additional** **file** **4.** **Supplemental Figure 3.** A survival figure related to the MELD-ΔLA score.

**Additional** **file** **5.** **Supplemental Figure 4.** The calibration curves of MELD-ΔLA score.

## Data Availability

The datasets generated and/or analyzed during the current study are available in the MIMIC-III repository, https://mimic.mit.edu/.

## Abbreviations

ICU: Intensive care unit
SOFA: Sequential organ failure assessment
MELD: Model for end-stage liver disease
INR: International normalized ratio
ΔLA: Decrease in lactate clearance
MELD-ΔLA: Mortality risk in critically ill cirrhotic patients
MIMIC-III: Medical Information Mart for Intensive Care
ACLF: Acute chronic liver failure
SBP: Systolic blood pressure
CLIF-C ACLF: Chronic liver failure consortium acute-on-chronic liver failure
CLIF-C AD: Chronic liver failure consortium-acute decompensation
DBP: Diastolic blood pressure
MAP: Mean arterial pressure
INR: International normalized ratio
PT: Prothrombin time
WBC: White blood cell count
PLT: Platelet count
Mean ± SE: Mean ± standard error
M (Q1, Q3): Median and quartile spacing
ROC: Receiver operator characteristic curve
HR: Hazard ratio
CI: Confidence interval
