# Supplementary figures and images for: Establishment of MELD-lactate clearance scoring system in predicting death risk of critically ill cirrhotic patients
Source: BMC Gastroenterol. 2022 Jun 3;22:280. doi: 10.1186/s12876-022-02351-5 (PMC9164412; doi:10.1186/s12876-022-02351-5)

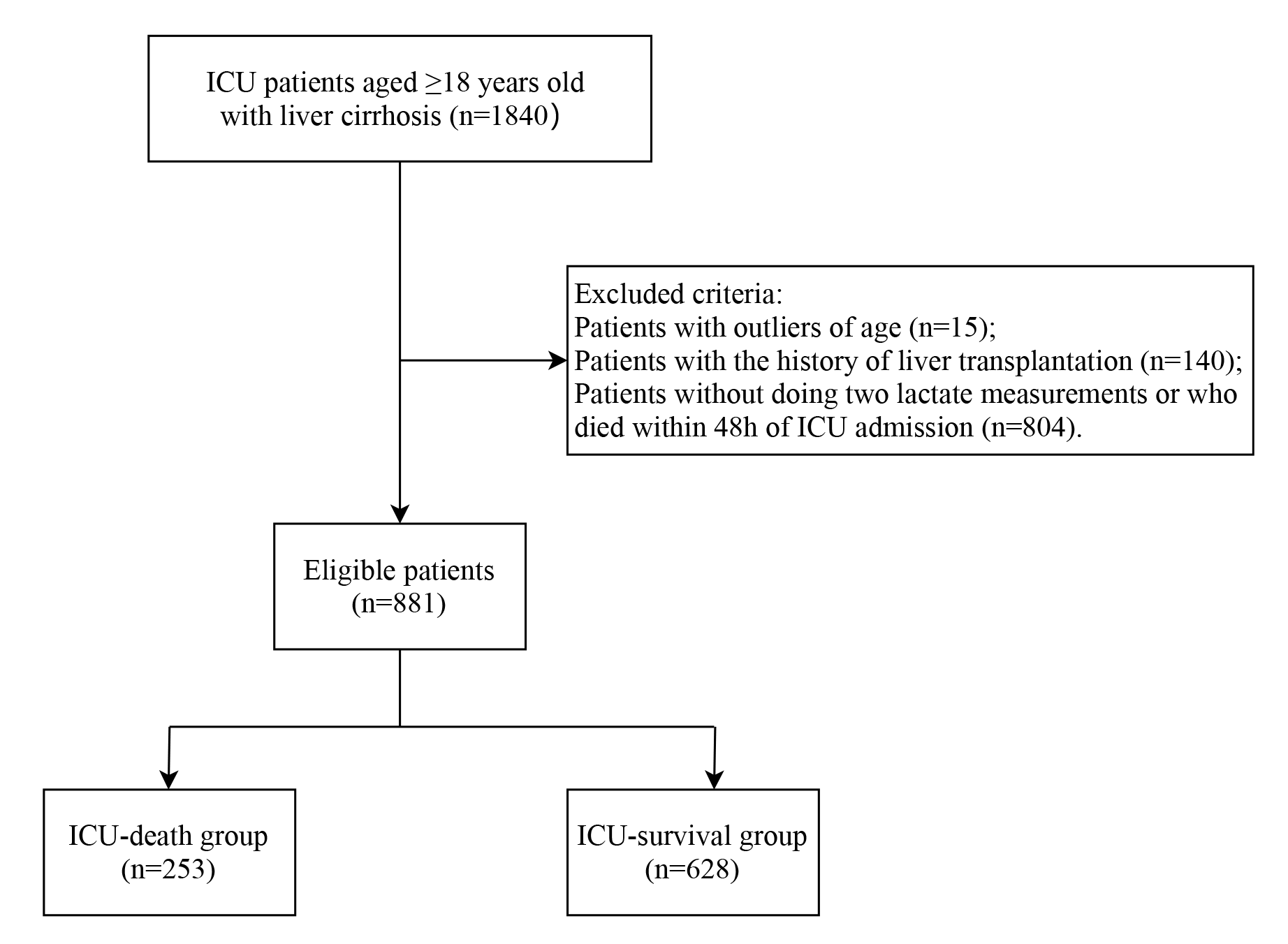

Supplement: Supplementary file 1 — Additional file 1. Supplemental Figure 1. The consort figure of extracted patient. [file 12876_2022_2351_MOESM1_ESM.tif]

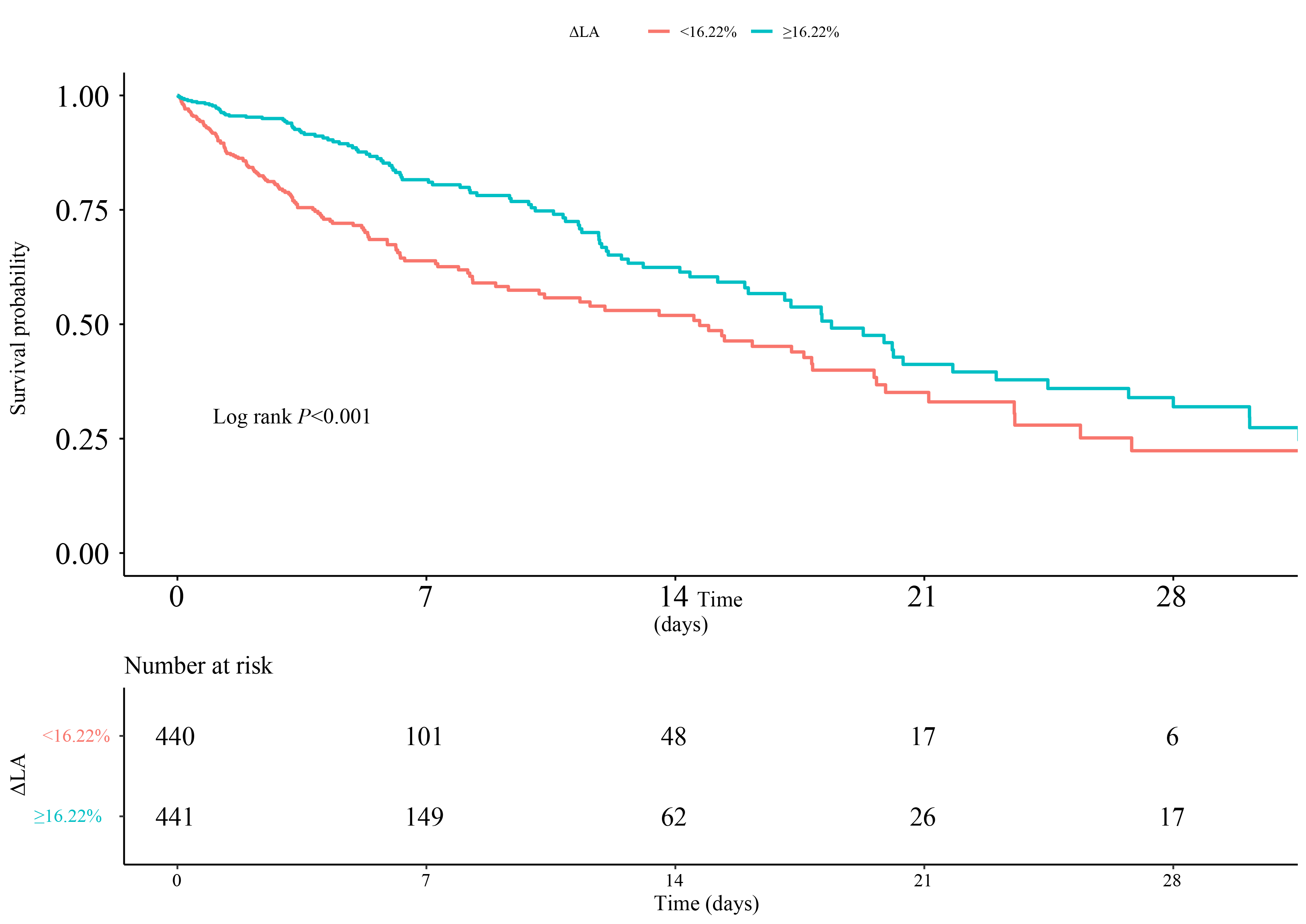

Supplement: Supplementary file 3 — Additional file 3. Supplemental Figure 2. The survival curves dependent on the lactate changes. [file 12876_2022_2351_MOESM3_ESM.tif]

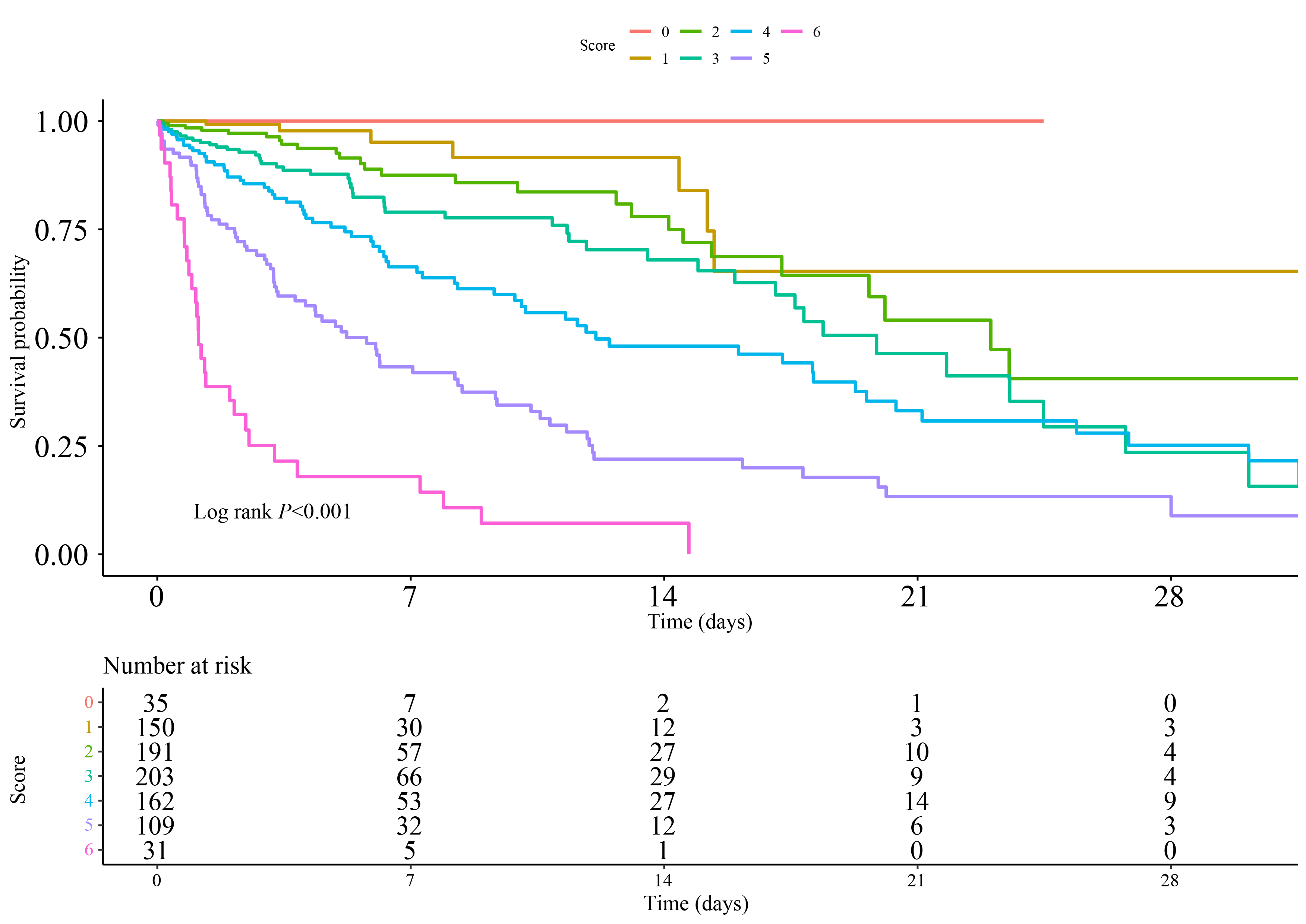

Supplement: Supplementary file 4 — Additional file 4. Supplemental Figure 3. A survival figure related to the MELD-ΔLA score. [file 12876_2022_2351_MOESM4_ESM.tif]

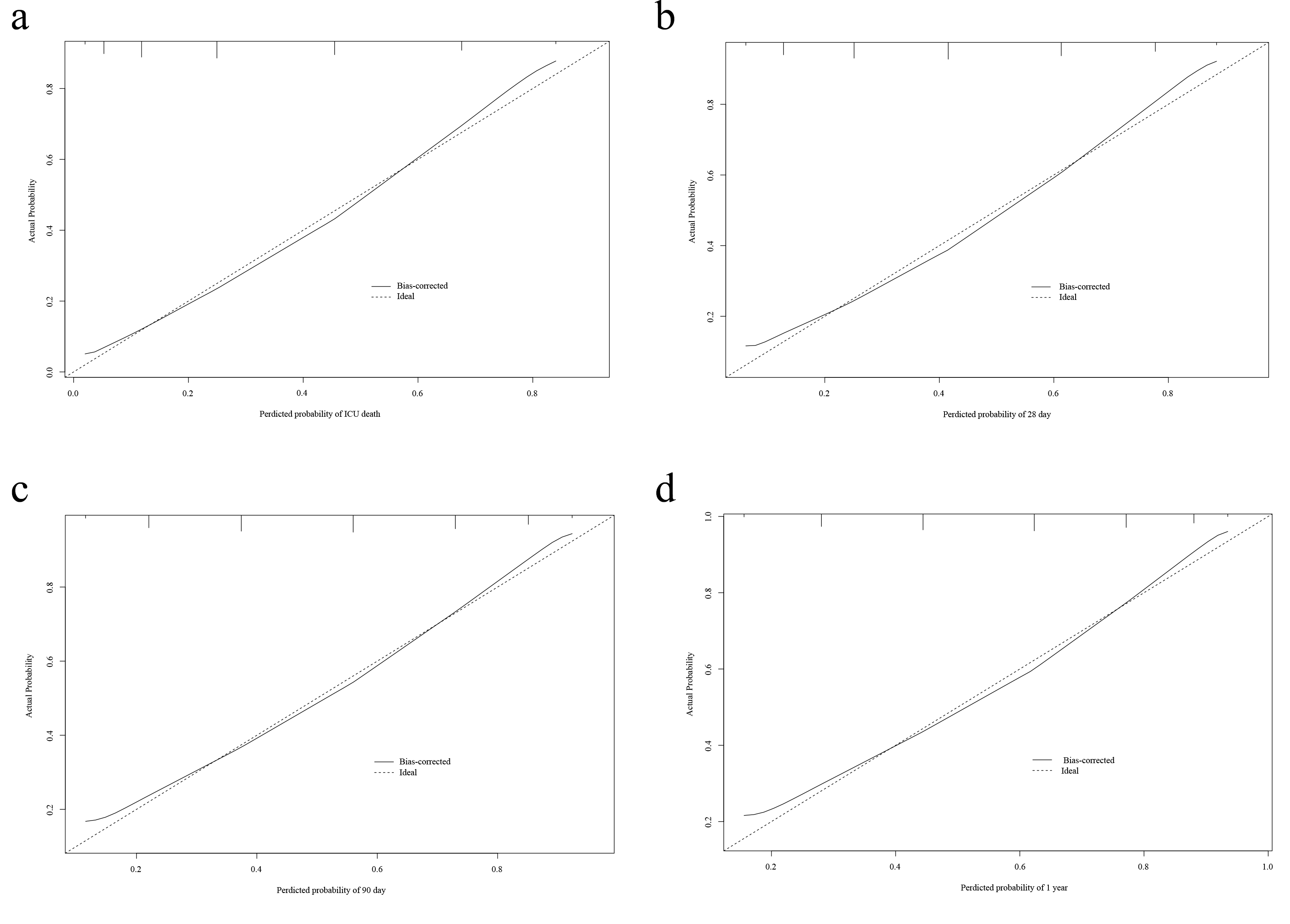

Supplement: Supplementary file 5 — Additional file 5. Supplemental Figure 4. The calibration curves of MELD-ΔLA score. [file 12876_2022_2351_MOESM5_ESM.tif]
