# Supplementary material for: Establishment of MELD-lactate clearance scoring system in predicting death risk of critically ill cirrhotic patients
Source: BMC Gastroenterol. 2022 Jun 3;22:280. doi: 10.1186/s12876-022-02351-5 (PMC9164412; doi:10.1186/s12876-022-02351-5)
Supplement: Supplementary file 2 — Additional file 2. Supplemental Table 1. The sensitivity analysis of missing data before and after interpolation. [file 12876_2022_2351_MOESM2_ESM.docx]

**Supplemental Table 1 The sensitivity analysis of missing data before and after interpolation**

| Variables | Ratio of missing values (%) | After the interpolation | Before the interpolation | Statistics | *P* |
| --- | --- | --- | --- | --- | --- |
| PaO_2_/FiO_2_, M (Q_1_, Q_3_) | 26.5 | 278.00 (170.00,395.00) | 285.00 (178.00,402.00) | Z=1.228 | 0.220 |
| FiO_2_, M (Q_1_, Q_3_) | 15.6 | 100.00 (50.00,100.00) | 100.00 (50.00,100.00) | Z=0.117 | 0.907 |
| Lymphocytes, M (Q_1_, Q_3_) | 12.7 | 10.40 (6.10,17.60) | 10.30 (6.00,17.40) | Z=-0.329 | 0.742 |
| Neutrophil, Mean ± SD | 12.7 | 76.62 ± 13.32 | 76.58 ± 13.60 | t=0.060 | 0.951 |
| PH, Mean ± SD | 2.8 | 7.36 ± 0.11 | 7.36 ± 0.11 | t=-0.070 | 0.941 |
| Race, n (%) | 9.1 |  |  | χ^2^=0.039 | 1.000 |
| White |  | 688 (78.09) | 624 (77.90) |  |  |
| Black |  | 76 (8.63) | 70 (8.74) |  |  |
| Asian |  | 26 (2.95) | 23 (2.87) |  |  |
| Hispanic |  | 48 (5.45) | 45 (5.62) |  |  |
| Others |  | 43 (4.88) | 39 (4.87) |  |  |
| Temperature, ℃, Mean ± SD | 7.3 | 36.66 ± 0.86 | 36.67 ± 0.86 | t=-0.420 | 0.673 |
| DBP, M (Q_1_, Q_3_) | 7.8 | 63.00 (53.00,72.00) | 62.00 (53.00,71.50) | Z=-0.263 | 0.793 |
| Heart rate, times/min, Mean ± SD | 4.0 | 94.90 ± 18.76 | 95.11 ± 18.75 | t=-0.240 | 0.812 |
| Albumin, Mean ± SD | 3.4 | 2.86 ± 0.66 | 2.85 ± 0.66 | t=0.220 | 0.829 |
| Marital status, n (%) | 4.6 |  |  | χ^2^=0.145 | 0.986 |
| Married |  | 389 (44.15) | 378 (45.00) |  |  |
| Single |  | 316 (35.87) | 295 (35.12) |  |  |
| Widowed |  | 52 (5.90) | 50 (5.95) |  |  |
| Divorced/Separated |  | 124 (14.07) | 117 (13.93) |  |  |
| Bilirubin, M (Q_1_, Q_3_) | 2.4 | 2.50 (1.20,6.20) | 2.60 (1.20,6.55) | Z=0.432 | 0.665 |
| Ventilation, n (%) | 1.2 | 369 (41.88) | 368 (42.30) | χ^2^=0.031 | 0.861 |
| MAP, Mean ± SD | 1.0 | 77.37 ± 17.96 | 77.41 ± 18.01 | t=-0.050 | 0.963 |
| SPO_2_, Mean ± SD | 1.0 | 96.38 ± 6.61 | 96.41 ± 6.62 | t=-0.110 | 0.916 |
| SOFA score, M (Q_1_, Q_3_) | 0.1 | 9.00 (7.00,12.00) | 9.00 (7.00,12.00) | Z=0.041 | 0.967 |

**Note:** PaO_2_: arterial oxygen partial pressure; FiO_2_: fraction of inspired oxygen; DBP: diastolic blood pressure; MAP: mean arterial pressure; SPO_2_: pulse oxygen saturation; SOFA: sequential organ failure assessment.
